# Incidence and management of myelosuppression in patients with chronic- and accelerated-phase chronic myeloid leukemia treated with omacetaxine mepesuccinate

**DOI:** 10.3109/10428194.2015.1071486

**Published:** 2015-10-05

**Authors:** Luke Akard, Hagop M. Kantarjian, Franck E. Nicolini, Meir Wetzler, Jeffrey H. Lipton, Michele Baccarani, H. Jean Khoury, Sandra Kurtin, Elizabeth Li, Mihaela Munteanu, Jorge Cortes

**Affiliations:** 1 Indiana Blood and Marrow Transplantation St. Francis Franciscan Alliance, Indianapolis, IN, USA; 2 The University of Texas MD Anderson Cancer Center, Houston, TX, USA; 3 Centre Hospitalier Lyon Sud, Pierre Bénite, and INSERM U1052, Centre de Recherche contre le Cancer de Lyon, Lyon, France; 4 Roswell Park Cancer Institute, Buffalo, NY, USA; 5 Princess Margaret Cancer Centre, Toronto, Ontario, Canada; 6 University of Bologna, Bologna, Italy; 7 Emory University School of Medicine, Atlanta, GA, USA; 8 University of Arizona Cancer Center, Tucson, AZ, USA; 9 PharmaStat LLC, Newark, CA, USA; 10 Teva Branded Pharmaceutical Products R&D, Frazer, PA, USA

**Keywords:** Hematologic toxicity, neutropenia, omacetaxine mepesuccinate, supportive care, thrombocytopenia

## Abstract

Omacetaxine mepesuccinate (Synribo^®^) is an inhibitor of protein synthesis indicated for the treatment of patients with chronic- or accelerated-phase chronic myeloid leukemia (CML) with resistance and/or intolerance to two or more tyrosine kinase inhibitors. Myelosuppression is the most common and clinically significant toxicity experienced by patients treated with omacetaxine. Here, we further examine the patterns of hematologic toxicity observed in clinical trials and describe the approach to management as well as resolution of events. Omacetaxine-related myelosuppression typically occurs more frequently during induction cycles. In general, the myelosuppression observed with omacetaxine treatment is manageable and reversible, and long-term administration is feasible. Careful monitoring, dose delays and reduction in administration days, and appropriate supportive care are critical for successful management of hematologic toxicity. Concerns regarding myelosuppression, observed with many cancer treatments, should not prevent eligible patients from receiving omacetaxine, particularly CML patients with unsatisfactory responses to multiple lines of prior treatment.

## Introduction

Chronic myeloid leukemia (CML) is distinguished by the presence of the *BCR-ABL* hybrid oncogene and expression of Bcr-Abl oncoprotein, which mediates the activation of signaling pathways leading to leukemogenesis [1]. Tyrosine kinase inhibitors (TKIs) that target Bcr-Abl represent the mainstay of CML treatment; however, for patients who develop resistance or intolerance to multiple TKIs, effective therapy remains a major unmetneed.

Omacetaxine mepesuccinate (omacetaxine – a semisynthetic form of homoharringtonine) is indicated for adult patients with chronic-phase CML (CML-CP) or accelerated-phase CML (CML-AP) with resistance and/or intolerance to two or more TKIs [2]. Omacetaxine is a transient inhibitor of protein synthesis and most profoundly impacts levels of short-lived proteins [3–6]. In preclinical studies, omacetaxine reduced levels of several oncoproteins, including Bcr-Abl and Mcl-1, and induced apoptosis in leukemic cells [7–10]. In clinical studies, omacetaxine produced durable hematologic and cytogenetic responses in patients with CML-CP and CML-AP, regardless of mutational status [11–15].

Among patients with CML-CP or CML-AP with resistance and/or intolerance to two or more TKIs, myelosuppression is the primary hematologic toxicity observed with omacetaxine treatment; in clinical studies, 85% of patients with CML-CP experienced grade ≥3 thrombocytopenia at any time during treatment, 81% experienced grade ≥3 neutropenia, and 62% experienced grade ≥3 anemia [2]. The majority of patients who received more than one cycle of treatment required at least one treatment delay, mainly due to myelosuppression. In spite of this toxicity, myelosuppression is typically manageable with a combination of treatment delays, reduced dosing days per cycle, supportive care, and patient education that allows for continuation of treatment. With the exception of myelosuppression-related events such as infection, grade 3/4 nonhematologic adverse events (AEs) were infrequent; most notably, grade 3 or 4 hyperglycemia was reported in 11% of patients [2,14,15]. Here, we further examine the hematologic toxicity in the safety population composed of all CML-CP and CML-AP patients with any prior TKI treatment and who received omacetaxine in the two omacetaxine clinical trials, and describe the monitoring, management, and outcome of these events to prescribing practitioners and healthcare professionals.

## Methods

### Patients

In a post hoc analysis, data were pooled from two multicenter, open-label, single-arm phase 2 studies (CML-202 and CML-203) in CML patients previously treated with TKIs [11,13,16]; data were included from all patients with CML-CP and CML-AP who participated in the two trials. Safety data were also included for four additional patients with CML-AP who were enrolled in a pilot study (CML-04.2/04.3) and were refractory to or had relapsed on imatinib (at a minimum).

### Treatment

Patients were treated with subcutaneous omacetaxine at 1.25 mg/m^2^ twice daily for up to 14 consecutive days per cycle. In the clinical trials (CML-202 and CML-203), patients received treatment for 14 consecutive days during induction (days 1–14 every 28 days until hematologic response), followed by maintenance omacetaxine given at 1.25 mg/m^2^ twice daily for 7 consecutive days every 28 days. If clinically indicated, hydroxyurea could be administered immediately before and during the first two cycles of treatment in patients with rapidly proliferating disease.

The omacetaxine dosing schedule was modified in the event of hematologic toxicity. For patients with grade 4 neutropenia (absolute neutrophil count [ANC] ≤ 0.5 × 10^9^/L and/or grade 3 thrombocytopenia (platelet count ≤50 × 10^9^/L), induction dosing was delayed until recovery to ANC ≥ 1.0 × 10^9^/L and platelet count ≥50 × 10^9^/L. Upon recovery, the number of dosing days per cycle was reduced by 2 days. Further reduction in the number of administration days per cycle (in decrements of 2 days) was allowed for patients with recurrent myelosuppression. Additionally, omacetaxine could be discontinued in patients with moderate to severe AEs considered possibly or probably related to treatment based on clinical investigator assessment. Administration of hematopoietic growth factors was allowed in the event of febrile neutropenia and use of erythropoietin or darbepoetin alfa was permitted for treatment of anemia. Patients did not receive prophylactic antibiotics.

### Assessments

The objective of this post hoc analysis was to characterize the incidence and duration of myelosuppression as well as its management in patients with CML-CP and CML-AP receiving omacetaxine therapy. In each of the clinical trials included, patients were assessed for safety and tolerability every 7 days during induction cycles and every 14 days during maintenance. The severity of myelosuppression events was graded according to the National Cancer Institute Common Terminology Criteria for Adverse Events, version 3.0. Hematologic nadirs were calculated as mean days to the lowest value in a cycle. To evaluate recovery from nadir, recovery thresholds were defined as platelets ≥50 × 10^9^/L, neutrophils ≥0.5 × 10^9^/L, and hemoglobin ≥80 g/L. In patients with nadirs below the threshold, time to recovery was defined as the number of days from nadir to recovery above threshold; patients without recovery were censored at the start of the next treatment cycle, or treatment discontinuation, whichever occurred first.

For patients with CML-CP, complete hematologic response (CHR) was defined as white blood cell count <10 × 10^9^/L, platelets<450 × 10^9^/L, myelocytes + metamyelocytes<5% in blood, no blasts or promyelocytes in blood, <20% basophils in peripheral blood, no extramedullary disease, and lasting ≥8 weeks. For CML-AP, CHR was defined as ANC ≥1.5 × 10^9^/L, platelets ≥100 × 10^9^/L, no blood blasts, bone marrow blasts <5%, no extramedullary disease, and lasting ≥4 weeks.

### Statistics

Descriptive statistics (number of patients, mean, median, range and standard deviation) were used to summarize continuous variables. For categorical variables, the number and percentage of total were tabulated. Median time to recovery and 95% confidence interval (CI) were obtained using a Kaplan–Meier estimate.

### Role of the funding source

The original research was sponsored by ChemGenex Pharmaceuticals Limited, Menlo Park, CA, USA (now a wholly owned subsidiary of Teva Branded Pharmaceutical Products R&D, Inc. Frazer, PA, USA). Teva Branded Pharmaceutical Products R&D provided financial support for medical writing and editorial review in preparation for submission for publication.

## Results

### Patients

A total of 108 patients with CML-CP and 55 patients with CML-AP were treated with subcutaneous omacetaxine at 1.25 mg/m^2^ twice daily. Patient demographics and baseline characteristics, including the incidence of myelosuppression at baseline are described in Table I. The median time from CML diagnosis to omacetaxine treatment was 63 months (range 8–234 months) for patients with CML-CP and 91 months (range 20–286 months) for those with CML-AP. Most patients had received two or more prior TKIs; 36 (33%) patients with CML-CP and 24 (44%) with CML-AP demonstrated resistance or intolerance to three TKIs (imatinib, dasatinib, and nilotinib). Twenty-four (22%) CML-CP and 14 (25%) CML-AP patients had received only one prior TKI (imatinib); these patients had a history of T315I.

### Exposure

A median of two (range 1–6) omacetaxine induction cycles were administered in both the CML-CP and CML-AP groups [Table II]. Patients with CML-CP received a median of eight (range 1–55) maintenance cycles and patients with CML-AP received a median of four (range 1–24) maintenance treatment cycles.

Ten patients (six CML-CP, four CML-AP; 8.6%) discontinued treatment due to hematologic toxicity: thrombocytopenia (two CML-CP, three CML-AP), pancytopenia (two CML-CP, one CML-AP), and aplasia and bone marrow necrosis (one CML-CP each). Most of these discontinuations occurred during early treatment cycles (cycle 1: *n*=7; cycle 2: *n*=4; cycle 3: *n*=1; cycle 7: *n*=2). In addition, one CML-CP patient and two CML-AP patients discontinued treatment due to infection.

### Incidence of myelosuppression

**Figure 1:**
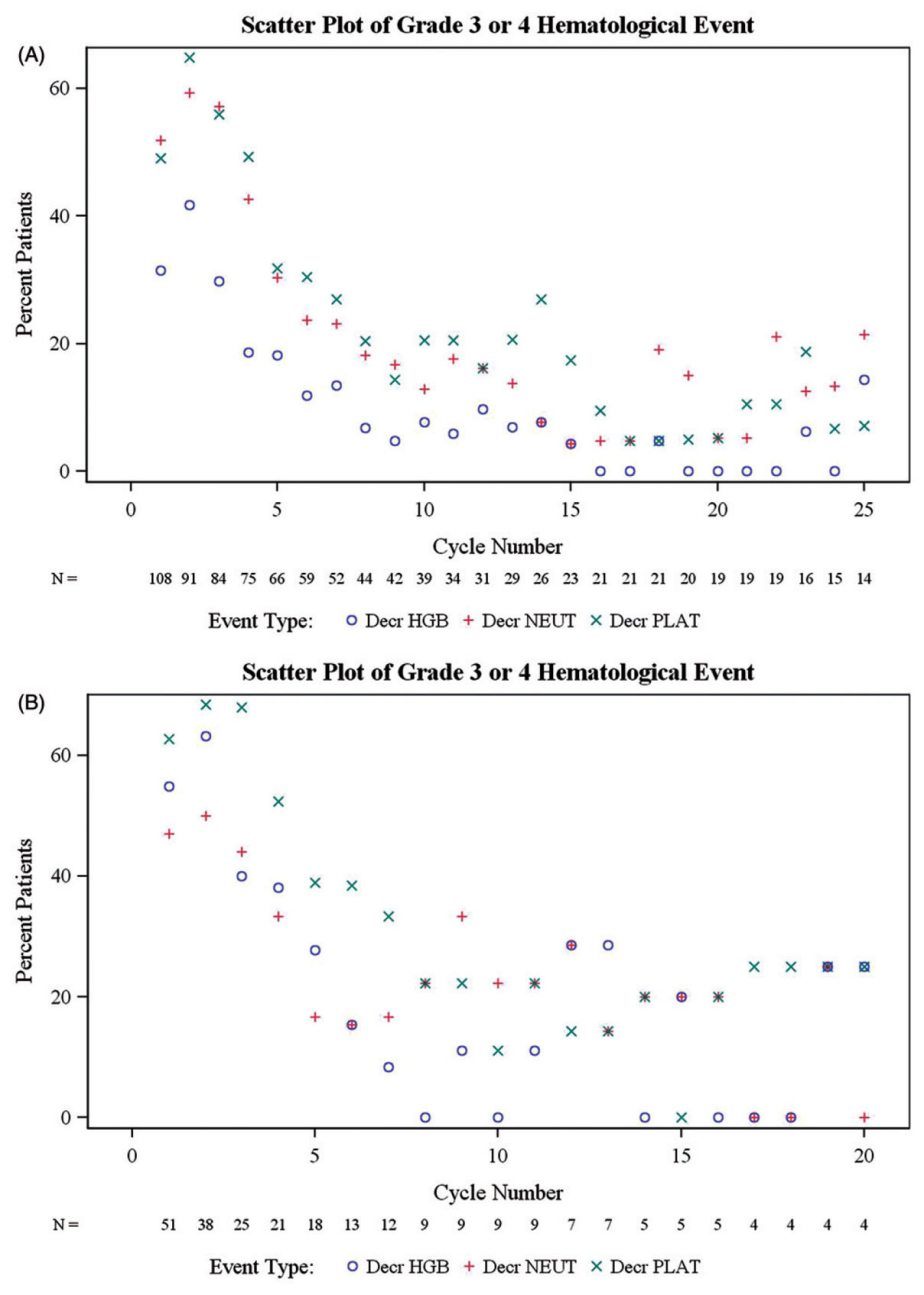
Proportion of chronic-phase patients (A) and accelerated-phase patients (B) experiencing grade 3/4 hematologic toxicity (anemia, neutropenia, and thrombocytopenia) by cycle.

The most frequent hematologic events were thrombocytopenia and anemia, and most events were grade ≥ 3 in severity [Table III]. The incidence of grade 3 or 4 hematologic toxicity (anemia, neutropenia, and thrombocytopenia) was highest within the first five cycles of treatment in patients with CML-CP [Fig. 1]. Starting counts and nadirs for platelets and neutrophils in CML-CP patients were lowest in cycles 3–6 [Table IV]. Platelet values recovered in a median of 13–28 days, neutrophil values 6–8 days, and hemoglobin values 4–13 days, during the first six cycles [Table IV]. For patients with CML-AP, median times to recovery of platelets, neutrophils, and hemoglobin were 2–7 days, 7–8 days, and 7–14 days, respectively, during the first four cycles. Rates of hematologic toxicity tended to be similar among CML-AP patients with or without major hematologic response (MHR; *n*=16 and 35, respectively), and were slightly higher in CML-CP patients with major cytogenetic response (MCyR; *n*=24) or MHR (*n*=79) vs. non-responders [Table V]. The incidence of myelosuppression was not higher in more heavily pretreated patients; in cycle 1 of treatment, for example, rates of grade 3/4 myelosuppression were 67%, 63%, and 47% in CP patients and 80%, 71%, and 79% in AP patients with 1, 2 or ≥ 3 prior TKIs, respectively.

### Myelosuppression-related events

Thirty-six CML-CP and 17 CML-AP patients experienced at least one serious myelosuppressive AE. Serious myelosuppression-related events included thrombocytopenia (10% CML-CP, 9% CML-AP patients), febrile neutropenia (6% CML-CP, 18% CML-AP), and anemia (7% CML-AP). In patients with CML-CP and CML-AP, eight and six events, respectively, of grade 3 or 4 hemorrhage occurred. Grade ≥ 3 bleeding events occurring in more than one patient included fatal cerebral hemorrhage in CML-CP and CML-AP patients (two each), and gastrointestinal hemorrhage in three CML-CP patients. Infection was reported in 49% and 56% of patients with CML-CP and CML-AP, respectively. Eleven grade 3/4 infections considered to be related to omacetaxine occurred in six CP patients (four pneumonias and one [each] extremity abscess, fungal infection, influenza, injection site abscess, sepsis, and urinary tract infection) and one AP patient (one pneumonia).

Five patients (5%) died from events related to myelosuppression that were considered to be related to omacetaxine by the investigator. The deaths of three CML-CP patients were attributed to sepsis (*n*=2) and pancytopenia (*n*=1). In two CML-AP patients, death was related to pancytopenia (*n*=1) and febrile neutropenia (*n*=1).

### Monitoring and management

Blood counts were typically obtained on a weekly basis during induction cycles; over 75% of patients in both disease phases underwent three or more scheduled blood draws per cycle in the first two cycles. During maintenance, monitoring was performed less frequently in many patients. Approximately 55% of patients with CML-CP underwent two or fewer scheduled blood draws per cycle after cycle 2. The proportion of patients with CML-AP who underwent three or more blood draws decreased from over 80% in cycles 1 and 2 to 56% and 67% in cycles 3 and 4, respectively. After cycle 4, 50% or more patients with CML-AP received two or fewer blood draws per cycle.

In general, hematologic toxicity was managed by treatment delays, reduction in the number of dosing days per cycle, and supportive care. The majority of CML-CP patients (87%) required at least one cycle delay, with a median of three cycle delays per patient (range 0–39 cycles) [Table II]. In CML-CP patients who received at least two cycles, the need for cycle delay days was greatest for cycle 2 (54% of patients experienced a median delay of 17 days [range 2–109]), cycle 3 (67%; median delay 25 days [range 2–184]), and cycle 4 (71%; median delay 13 days [range 1–81]). Fewer CML-AP patients required cycle delays (68%), with a median of one cycle delay per patient (range 0–19 cycles). In AP patients, the cycle delay days were greatest for cycle 3 (44% of patients experienced a median delay of 31 days [range 4–75]), cycle 4 (67%; median delay 14 days [range 1–74]), and cycle 5 (44%; median delay 17 days [range 3–46]). In both CP and AP patients, thrombocytopenia was the most frequent reason for cycle delay, followed by neutropenia and pancytopenia. Approximately half of CML-CP (56%) and CML-AP (47%) patients required a reduction in the number of dosing days per cycle (for any reason) during induction; during maintenance cycles, 75% and 64%, respectively, required a reduced number of dosing days [Table II].

**Figure 2:**
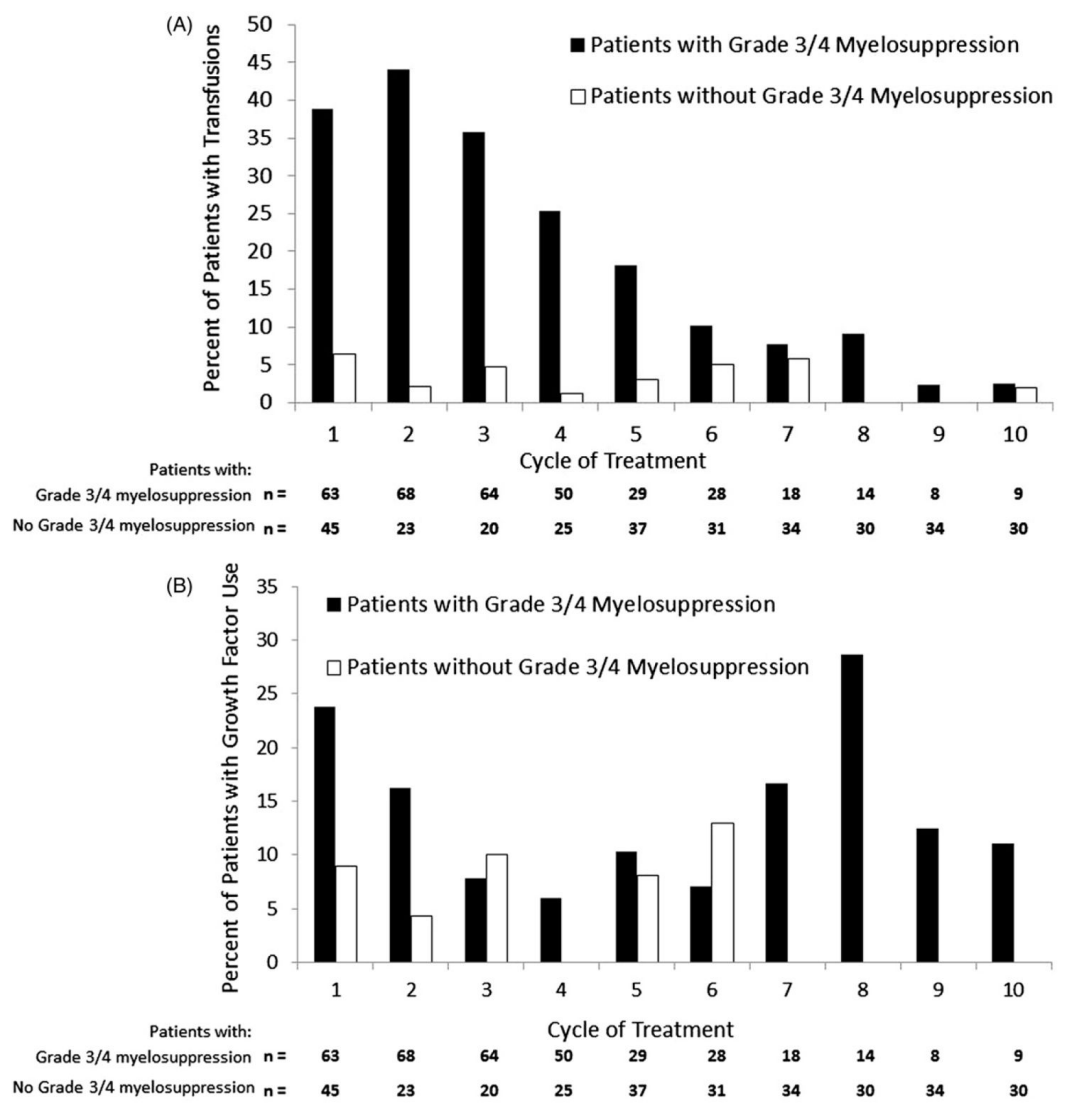
Transfusions (A) and growth factor use (B) by treatment cycle in CML-CP patients (*n* = 108) with or without grade 3/4 myelosuppression. Percentages are based on the number of patients in each category. Grade 3 or 4 myelosuppression is defined as any grade 3 or 4 neutropenia, thrombocytopenia, and/or anemia detected by laboratory tests. Patients are counted once in a given cycle if multiple myelosuppression events occurred.

The majority of patients in both disease phases received concomitant hematologic supportive care, consisting of transfusions and growth factors. Red blood cell transfusions were administered in 49% and 43% of CML-CP and CML-AP patients, respectively; platelets were administered in 56% and 51%. The median number of transfusions administered in CML-CP patients (among patients who received transfusions) was four (range 1–19) for platelets and three (range 1–55) for red blood cells; in CML-AP patients the median number of transfusions per patient was three (range 1–40) for platelets and four (range 1–18) for red blood cells. In the CML-CP population, use of transfusions was highest in patients with grade 3/4 myelosuppression and within the first five cycles of treatment [Fig. 2]; a similar trend was observed in CML-AP patients (data not shown).

Twenty-five percent of patients with CML-CP and 10% with CML-AP received concomitant granulocyte colony-stimulating factors. Erythropoiesis-stimulating agents were administered to 22% of CML-CP and 14% of CML-AP patients. The incidence of growth factor use was generally similar in early and later cycles [Fig. 2].

In patients with CML-CP, the proportion of patients receiving transfusions decreased in later cycles, from 46–27% in cycles 1–4, 21–14% in cycles 5–7, and<10% in cycles 8–10. In patients with CML-AP, the proportion of patients receiving transfusions decreased in later cycles, from 63–24% in cycles 1–4, 22–8% in cycles 5–7, and approximately 11% in cycles 8–10. Rates of transfusions were slightly higher in patients with dose delays (vs. those without), and in CML-CP patients with dose reductions during induction (vs. those without) [Table VI]. Having a CHR at the start of therapy with omacetaxine did not influence the need for transfusions: 76% of CML-CP patients (73% of CML-AP patients) with CHR at baseline were transfused vs. 73% of CML-CP patients (83% of CML-AP patients) without CHR at baseline [Table VII].

### Resolution of events

Among patients with CML-CP, most grade 3/4 thrombocytopenia (91%), neutropenia (96%), and anemia (91%) resolved with supportive care [Table VIII]. Notably, neutrophil and hemoglobin levels reached recovery threshold levels (neutrophils ≥0.5 × 10^9^/L, and hemoglobin ≥80 g/L) at approximately 2 weeks, while recovery of platelet levels (platelets ≥50 × 10^9^/L) occurred at approximately 3 weeks. Similarly, among patients with CML-AP, nearly all grade 3/4 thrombocytopenia (88%), neutropenia (100%), and anemia (93%) resolved, but recovery of neutrophils (approximately 2 weeks) lagged behind platelet and hemoglobin recovery (approximately 1 week) [Table VIII].

## Discussion

Myelosuppression is among the most clinically important toxicities associated with the treatment of CML patients demonstrating treatment resistance or intolerance. For instance, among patients treated with the TKI bosutinib in the third-line setting, 41% experienced grade 3 or 4 hematologic toxicity (26% thrombocytopenia, 15% neutropenia, 7% anemia), leading to treatment interruption in 46% of affected patients and dose reduction in 32% [17]. Among patients treated with ponatinib (over 90% of whom had received at least two prior TKIs), 37% experienced any grade thrombocytopenia, 19% experienced neutropenia, and 9% experienced anemia; the majority of events were grade 3 or 4 [18].

Myelosuppression occurs in the majority of omacetaxine-treated patients and at a slightly higher rate among CML-CP patients with cytogenetic or hematologic response vs. non-responders [Table V]. Despite this significant toxicity, omacetaxine treatment is feasible in many CML-CP and CML-AP patients with CML, inducing hematologic and cytogenetic responses in some patients who are able to continue treatment. In a post hoc analysis of the efficacy population from the two phase 2 studies, 11 of 50 patients (22%) with CML-CP who completed more than three cycles of omacetaxine achieved MCyR; three of these patients maintained a response for at least 12 months [15]. Rate of CHR was 94% in this subgroup and the response was durable (≥12 months) in 26% [15]. Progression-free survival (PFS) and overall survival (OS) of 9.9 months and 49.3 months, respectively, were associated with patients who received more than three cycles of treatment. In the efficacy population (*n*=76), PFS and OS were 9.6 months and 40.3 months, respectively. Among 14 patients with CML-AP who received more than three cycles of omacetaxine treatment, MHR was achieved in four patients (29%). PFS and OS were 7 months and 24.6 months, respectively, in patients who received more than three cycles of treatment, and 3.6 months and 14.3 months in the overall population (*n*=35) [15]. While this subanalysis showed that the majority of responses in patients with CML-CP occurred within the first three cycles, two patients achieved MCyR after cycle 3 and complete cytogenetic response may occur as late as 8.5 months [15]. Thus, effective management of myelosuppression, including withholding treatment and reducing the number of days dosed per cycle, and early use of growth factors and transfusions, is essential to allow continuation of treatment; this is particularly important during the early induction cycles (cycles 1–4), in which the rates of grade 3/4 hematologic toxicities were highest.

Delay of treatment until recovery of blood counts is imperative in patients with myelosuppression; once recovery has occurred, a reduction in the number of dosing days in subsequent cycles will often allow continued treatment. Both dose delays and reductions are encouraged if warranted at any point throughout the treatment period. In addition, avoidance of anticoagulants, aspirin, and nonsteroidal anti-inflammatory drugs when the platelet count is <50,000/μL is necessary as this may increase the risk of bleeding. In this clinical trial experience, the need for supportive care, particularly transfusions, may be highest in early cycles (cycles 1–4), with fewer than 10% of patients requiring transfusions in maintenance cycles.

Careful monitoring of patients for myelosuppression is necessary. Complete blood counts should be performed weekly during induction and initial maintenance cycles and every 2 weeks during later maintenance cycles, as clinically indicated. Patients with treatment-related neutropenia should be watched for signs of infection (e.g. fever) and be instructed to contact their physician immediately in case of signs or symptoms of infection. Patients should be educated and given home training materials by their treating physician or oncology nurse regarding important signs and symptoms suggestive of hemorrhage (unusual bleeding, easy bruising, or blood in urine or stool; confusion, slurred speech, or altered vision) or to bring to the physician’s attention if they plan to have any dental or surgical procedures [19,20]. Equally important is to be aware of any concomitant medications that may increase the risk of bleeding. Working closely with oncology providers such as the oncology nurse can minimize patient- and environment-related sources of infection and increase awareness of signs and symptoms of bleeding during home administration of omacetaxine or between visits to the clinic.

In these trials, few patients required discontinuation of treatment due to hematologic toxicity, and persistent toxicity was rare, with most events resolved within 1–2 weeks during the early treatment cycles. Hematologic toxicity was not cumulative with continued treatment; rather, rates of grade ≥3 hematologic toxicities were reduced in later cycles, congruent with a switch to maintenance dosing. However, toxicities experienced in early cycles led to study discontinuation in some patients, thus possibly introducing a bias in patient selection. Future studies exploring alternative treatment schedules are warranted to examine whether myelosuppression (and all resulting sequelae) can be minimized.

## Conclusions

Myelosuppression should be anticipated in patients with CML who are treated with omacetaxine. However, concerns regarding toxicity should not prevent omacetaxine administration as some patients could experience durable cytogenetic or hematologic responses with continued treatment. In heavily pretreated patients, frequent monitoring of laboratory parameters, use of adequate dose modifications, adequate patient training for signs and symptoms, and appropriate medical support are critical for the successful management of hematologic toxicity and continued treatment. For patients initiating omacetaxine, provision of informational pamphlets and maintaining close communication with oncology care providers (e.g. follow-up by phone) are important, particularly for patients who live far from the treatment center.

## Figures and Tables

**Table I:** Patient baseline characteristics and demographics.

	Chronic phase (*n* = 108)	Accelerated phase (*n* = 55)
Median age (range), years	58.0 (20.0–83.0)	57.0 (23.0–83.0)
≥65 years, *n* (%)*	29 (27)	21 (38)
>75 years, *n* (%)	5 (5)	2 (4)
Sex, *n* (*%*)		
Male	68 (63)	34 (62)
Female	40 (37)	21 (38)
Race, *n* (*%*)		
White	79 (73)	29 (53)
Black or African American	6 (6)	10 (18)
Hispanic	3 (3)	2 (4)
Asian	15 (14)	9 (16)
Other or unknown	5 (5)	5 (9)
ECOG performance status, *n* (*%*)		
0	68 (63)	13 (26)†
1	37 (34)	30 (59)†
≥2	3 (2.8)	8 (16)†
NYHA classification, *n* (*%*)		
I	105 (97)	47 (92)†
II	3 (3)	2 (4)†
Median time from CML diagnosis to omacetaxine treatment, months (range)	63.0 (7.9–234.3)	91.4 (20.3–285.6)†
CHR at baseline, *n* (*%*)‡	29 (27)	11 (22)†
Previous TKI treatment		
Imatinib	108 (100)	51 (100)†
Dasatinib	72 (67)	38 (75)†
Nilotinib	48 (44)	27 (53)†
Other antineoplastic agents§	13 (12)	9 (18)†
Resistance/intolerance to previous TKI treatment, *n* (*%*)¶		
Imatinib only	24 (22)	14 (25)
Imatinib and dasatinib	36 (33)	14 (26)
Imatinib and nilotinib	12 (11)	3 (6)
Imatinib, dasatinib, and nilotinib	36 (33)	24 (44)
Previous hydroxyurea use, *n* (*%*)||	54 (50)	26 (51)†
Baseline myelosuppression, *n* (*%*)		
*Thrombocytopenia*		
*n*	107	51
Any grade ≥1	15 (14)	27 (53)
Grade 3 or 4	1 (1)	15 (30)
*Neutropenia*		
*n*	106	51
Any grade ≥1	18 (17)	17 (33)
Grade 3 or 4	1 (1)	8 (16)
*Anemia*		
*n*	107	51
Any grade ≥1	59 (55)	40 (78)
Grade 3 or 4	4 (4)	3 (6)

CHR, complete hematologic response.

CML, chronic myeloid leukemia.

ECOG, Eastern Cooperative Oncology Group.

NYHA, New York Heart Association.

TKI, tyrosine kinase inhibitor.

* Includes patients >75 years of age.

† Data available for 51 of 55 CML-AP patients.

‡ Based on data monitoring committee (DMC) adjudicated results. If DMC-adjudicated CHR status was not available, the assessment from study site principal investigator was used. In patients with CML-CP, CHR was defined as white blood cell count <10 × 10^9^/L, platelets <450 × 10^9^/L, myelocytes + metamyelocytes <5% in blood, no blasts or promyelocytes in blood, <20% basophils in peripheral blood, and no extramedullary disease. For CML-AP, CHR was defined as absolute neutrophil count ≥1.5 × 10^9^/L, platelets ≥100 × 10^9^/L, no blood blasts, bone marrow blasts <5%, and no extramedullary disease.

§ Certain investigational TKIs were coded as “Other antineoplastic agents.”

¶ Includes patients who received one or more approved TKI, regardless of chemotherapy, interferon, or investigational TKI.

|| Given within 48 hours of the last predose laboratory assessment.

**Table II:** Exposure and dose delays.

Exposure	Chronic phase	Accelerated phase
Number of patients	108	55
Received cycles, median (range)	6 (1–58)	2 (1–29)
Treatment days per cycle, median (range)	9 (1–15)	13 (1–17)
Patients with ≥ one cycle delay*, *n* (%)	79 (87)	27 (68)
Cycle delays per patient, median (range)	3 (0–39)	1 (0–19)
Induction cycles†		
Number of patients	108	55†
Received cycles, median (range)	2 (1–6)	2 (1–6)
Median number of cycle reductions per patient (range)	1 (0–5)	0 (0–3)
Patients with ≥ one cycle of dose reduction‡, *n* (%)	60 (56)	24 (47)
Patients without any dose reduction, *n* (%)	48 (44)	27 (52)
Maintenance cycles†		
Number of patients	68	22
Received cycles, median (range)	8 (1–49)	4 (1–24)
Median number of cycle reductions per patient (range)	4 (0–54)	1 (0–23)
Patients with ≥ one cycle of dose reduction‡, *n* (%)	51 (75)	14 (64)
Patients without any dose reduction, *n* (%)	17 (25)	8 (36)

* Percent calculated based on the number of patients with two or more treatment cycles.

† Data available for 51 of 55 accelerated-phase patients.

‡ Dose reduction is the reduction in number of days dosed (<14 dosing days for induction cycles; <7 dosing days for maintenance cycles).

**Table III:** Hematologic treatment-emergent adverse events occurring in ≥5% of patients with CML-CP and CML-AP.

Event, *n* (%)	Chronic phase (*n* = 108)		Accelerated phase (*n* = 55)	
	
	Any grade	Grade ≥ 3	Any grade	Grade ≥ 3
Thrombocytopenia	82 (76)	73 (68)	32 (58)	27 (49)
Anemia	66 (61)	39 (36)	28 (51)	21 (38)
Neutropenia	56 (52)	50 (46)	11 (20)	10 (18)
Leukopenia	23 (21)	20 (19)	6 (11)	3 (6)
Lymphopenia	18 (17)	17 (16)	1 (2)	1 (2)
Bone marrow failure*	11 (10)	11 (10)	0	0
Febrile neutropenia	11 (10)	11 (10)	11 (20)	9 (16)
Pancytopenia	10 (9)	8 (7)	3 (5)	3 (5)
Leukocytosis	5 (5)	1 (1)	6 (11)	4 (7)

AP, accelerated phase

CML, chronic myeloid leukemia

CP, chronic phase.

* Includes verbatim term “myelosuppression.”

**Table IV:** Hematologic parameters by cycle (1–10) in patients with CML-CP: starting and nadir* values, time to nadir, and time to recovery†,‡.

	Platelet counts × 10^9^/L (median)				Neutrophil counts × 10^9^/L (median)				Hemoglobin g/L (median)			
		
	Start (range)	Nadir (range)	Time to nadir days (95% CI)	Time to recovery days (95% CI)	Start (range)	Nadir (range)	Time to nadir days (95% CI)	Time to recovery days (95% CI)	Start (range)	Nadir (range)	Time to nadir days (95% CI)	Time to recovery days (95% CI)
Cycle 1, *n* = 75	177 (0.4–1127)	51 (0.1–825)	21 (20–22)	15 (10–32)	5.2 (0.1–142.7)	0.7 (0–12.8)	22 (21–23)	7 (5–14)	119 (72–155)	93 (54–139)	22 (21–23)	4 (2–6)
Cycle 2, *n* = 60	177.5 (29–877)	29.5 (0–636)	22 (20–25)	19 (11–42)	3.1 (0–28.6)	0.7 (0–5.7)	21 (18–24)	7 (7–9)	109 (11.4–153)	85 (9.4–150)	22 (18–24)	7 (3–9)
Cycle 3, *n* = 55	81 (28–464)	36 (0–457)	18 (15–22)	14 (11–22)	2.5 (0.7–58.3)	0.8 (0–10.5)	17 (15–21)	7 (5–13)	108 (62–142)	89 (9.1–133)	16 (14–21)	9 (4–18)
Cycle 4, *n* = 50	92 (15–711)	39 (1–711)	19.0 (15–22)	24 (13–32)	2.4 (0.7–15.6)	1.1 (0–6.8)	16 (15–22)	6.5 (1–20)	110 (71–151)	94.5 (11.4–151)	14 (13–16)	13 (1–14)
Cycle 5, *n* = 42	99 (35–1146)	58.5 (6–733)	15 (15–19)	28 (7–44)	2.5 (0.8–18.4)	1.4 (0.3–11.9)	15 (15–21)	8 (2–16)	112 (70–173)	103 (10.2–173)	15 (14–16)	7.5 (3.0–NA)
Cycle 6, *n* = 40	91.4 (34–739)	55.5 (2–739)	15 (13–17)	13 (6–36)	2.3 (0.9–24)	1.4 (0–7.7)	16 (12–22)	6 (5–7)	112.5 (67–166)	95.5 (64–162)	15.5 (14–19)	7 (NA)
Cycle 7, *n* = 36	96.5 (4.3–699)	72.5 (4.3–699)	15.5 (11–20)	13 (4–31)	2.4 (0.7–19.9)	1.7 (0–8.8)	16 (15–21)	NA	113 (63–148)	101.0 (63–148)	14.5 (13–16)	21.0 (7–NA)
Cycle 8, *n* = 30	104.5 (37–768)	93 (4–625)	16 (13–20)	19 (5–32)	3.1 (0.5–49.8)	2.1 (0–9.7)	18 (15–21)	29.5 (11–48)	121 (76–153)	113.5 (65–143)	14 (13–21)	13.5 (2–25)
Cycle 9, *n* = 28	110 (42–616)	98.5 (8–616)	13.5 (8–15)	14 (7–28)	3.2 (0.4–40.3)	2.1 (0–10.3)	15 (14–23)	NA	123 (73–145)	114 (62–145)	15 (14–21)	11 (7–15)
Cycle 10, *n* = 26	125.5 (27–494)	99 (17–494)	14.5 (7–19)	22 (6–NA)	2.5 (1.1–16)	1.9 (0.2–16)	15 (10–20)	NA	123 (68–136)	115 (68–136)	12.5 (9.0–15)	13 (7–NA)

CML, chronic myeloid leukemia

CP, chronic phase

NA, not available.

* Nadir is defined as the lowest value in a cycle.

† Threshold for recovery is defined as ≥ 50 × 10^9^/L for platelet count; >0.5 × 10^9^/L for neutrophil count; and ≥ 80 g/L (or ≥ 4.9 mmol/L) for hemoglobin.

‡ The time to recovery is defined as the number of days from date of nadir to date of recovery; patients without recovery were censored at the start of the next treatment cycle, or treatment discontinuation, whichever occurred first.

**Table V:** Rates of myelosuppression in CML-CP and CML-AP patients with and without major hematologic response (MHR), and major cytogenetic response (MCyR) to omacetaxine.

Blood and lymphatic system disorders, *n* (%)	MCyR Responders/nonresponders*			MHR Responders/nonresponders*					
	
	Chronic phase			Chronic phase			Accelerated phase		
*n*	24	/	84	79	/	29	16	/	35
Any	23 (96)	/	69 (82)	75 (94.9)	/	17 (59)	13 (81)	/	25 (71)
Anemia	18 (75)	/	48 (57)	55 (70)	/	11 (38)	9 (56)	/	18 (51)
Bone marrow failure†	4 (17)	/	7 (8)	10 (13)	/	1 (3)	0	/	0
Febrile neutropenia	3 (13)	/	8 (10)	11 (14)	/	0	5 (31)	/	4 (11)
Leukocytosis	0	/	5 (6)	4 (5)	/	1 (3)	2 (13)	/	4 (11)
Leukopenia	6 (25)	/	17 (20)	19 (24)	/	4 (14)	2 (13)	/	4 (11)
Lymphopenia	7 (29)	/	11 (13)	15 (19)	/	3 (10)	0	/	1 (3)
Neutropenia	19 (79)	/	38 (45)	50 (63)	/	7 (24)	6 (38)	/	5 (14)
Pancytopenia	2 (8)	/	8 (10)	7 (9)	/	3 (10)	2 (13)	/	1 (3)
Thrombocytopenia	20 (83)	/	62 (74)	70 (89)	/	12 (41)	10 (63)	/	21 (60)

AP, accelerated phase

CML, chronic myeloid leukemia

CP, chronic phase.

* Response data provided for safety population based on data monitoring committee (DMC)-adjudicated results. If DMC-adjudicated response was not available, the assessment from the study site principal investigator was used. MCyR defined as 0–35% Philadelphia chromosome-positive cells. In patients with CML-CP, MHR was defined as complete hematologic response (CHR). In patients with CML-AP, MHR was defined as CHR, no evidence of leukemia, or return to CML-CP.

† Includes verbatim term “myelosuppression.”

**Table VI:** Transfusion in CML-CP and CML-AP patients with or without dose delays.

	Chronic phase (*n* =108)			Accelerated phase (*n* = 51)		
Patients with/without dose delay, *n* (%)						
*n*	79	/	12	25	/	13
Received transfusion	64 (81)	/	7 (58)	21 (84)	/	9 (69)
Did not receive transfusion	15 (19)	/	5 (42)	4 (16)	/	4 (31)
Median (range) number of transfusions	9 (2–58)	/	3 (2–14)	6 (2–29)	/	2 (2–4)
Patients with/without a reduction in dosing days during induction*, *n* (%)						
*n*	60	/	48	24	/	27
Received transfusion	48 (80)	/	33 (69)	19 (79)	/	22 (82)
Did not receive transfusion	12 (20)	/	15 (31)	5 (21)	/	5 (19)
Median (range) number of transfusions	3 (1–6)	/	1 (1–6)	3 (1–6)	/	1 (1–6)
Patients with/without a reduction in dosing days during maintenance*, *n* (%)						
*n*	51	/	17	14	/	8
Received transfusion	40 (78)	/	13 (77)	10 (71)	/	6 (75)
Did not receive transfusion	11 (22)	/	4 (23)	4 (29)	/	2 (25)
Median (range) number of transfusions	10 (1–55)	/	3 (1–32)	4 (1–24)	/	3.5 (1–23)

AP, accelerated phase.

CML, chronic myeloid leukemia.

CP, chronic phase.

* Reduction in dosing days is defined as total dosing days <14 in any one cycle during induction or total dosing days <7 in any one cycle during maintenance.

**Table VII:** Transfusions in patients with CML-CP and CML-AP by complete hematologic response (CHR) at baseline (top), and hematologic response* to omacetaxine (bottom).

	Chronic phase (*n*=108)			Accelerated phase (*n*=51)		
CHR + at baseline/CHR – at baseline						
*n*	29	/	79	11	/	40
Blood and related products, *n* (%)	22 (76)	/	58 (73)	8 (73)	/	33 (83)
Blood†	2 (7)	/	7 (9)	1 (9)	/	3 (8)
Platelets‡	19 (66)	/	42 (53)	5 (46)	/	26 (65)
Red blood cells§	22 (76)	/	53 (67)	7 (64)	/	29 (73)
Blood substitutes and plasma protein fractions, *n* (%)	0	/	1 (1)	0	/	1 (3)
Plasma	0	/	1 (1)	0	/	1 (3)
Responders/non-responders*						
*n*	79	/	29	16	/	35
Blood and related products, *n* (%)	62 (79)	/	18 (62)	13 (81)	/	28 (80)
Blood†	7 (9)	/	2 (7)	1 (6)	/	3 (9)
Platelets‡	47 (60)	/	14 (48)	9 (56)	/	22 (63)
Red blood cells§	58 (73)	/	17 (59)	12 (75)	/	24 (69)
Blood substitutes and plasma protein fractions, *n* (%)	1 (1)	/	0	0	/	1 (3)
Plasma	1 (1)	/	0	0	/	1 (3)

AP, accelerated phase; CML, chronic myeloid leukemia; CP, chronic phase.

Note: Patients are counted once within a medication class and medication name. Transfusions started after the last study drug dosing or ended before the first study drug dosing are excluded from the summary.

* Response data provided for safety population based on data monitoring committee (DMC)-adjudicated results. If DMC-adjudicated response was not available, the assessment from the study site principal investigator was used. Hematologic response defined as complete hematologic response, no evidence of leukemia, or return to chronic-phase CML.

† Contains whole and packed human blood cells.

‡ Contains concentrated platelets.

§ Contains concentrated red blood cells.

**Table VIII:** Reversibility of hematologic treatment-emergent adverse events (by preferred term) occurring in ≥10% of patients with CML-CP or CML-AP.

	Chronic phase (*n* = 108)		Accelerated phase (*n* = 55)	
	
	Any grade	Grade ≥3	Any grade	Grade ≥3
Thrombocytopenia				
Patients reporting event, *n*	82	73	32	27
Events, *n*	300	216	100	83
Event duration, days, median (range)	21 (1–1233)	22 (1–1233)	8 (1–1105)	9 (1–1105)
Resolved events, *n*	276	197	90	73
Anemia				
Patients reporting event, *n*	66	39	28	21
Events, *n*	212	86	79	41
Event duration, days, median (range)	15 (1–1233)	12 (1–1233)	8 (1–721)	6 (1–159)
Resolved events, *n*	187	78	68	38
Neutropenia				
Patients reporting event, *n*	57	51	11	10
Events, *n*	187	135	36	32
Event duration, days, median (range)	14 (1–802)	14 (1–802)	15 (2–82)	15 (2–82)
Resolved events, *n*	182	130	36	32
Leukopenia				
Patients reporting event, *n*	23	20	6	3
Events, *n*	87	50	24	16
Event duration, days, median (range)	14 (1–156)	14 (2–148)	10 (2–738)	11 (2–128)
Resolved events, *n*	84	48	22	14
Lymphopenia				
Patients reporting event, *n*	18	17	1	1
Events, *n*	54	29	3	1
Event duration, days, median (range)	15 (2–702)	10 (2–125)	21 (15–57)	21 (21–21)
Resolved events, *n*	51	27	3	1
Bone marrow failure*				
Patients with events, *n*	11	11	0	0
Events, *n*	14	14		
Event duration, days, median (range)	26 (3–326)	26 (3–326)		
Resolved events, *n*	14	14	0	0
Febrile neutropenia				
Patients reporting event, *n*	11	11	11	9
Events, *n*	14	13	14	12
Event duration, days, median (range)	8 (3–33)	8 (3–33)	12 (2–32)	12 (2–32)
Resolved events, *n*	14	13	14	12
Fatal events, *n*	0	0	0	0
Pancytopenia				
Patients reporting event, *n*	10	8	3	3
Events, *n*	18	9	3	3
Event duration, days, median (range)	22 (2–246)	46 (2–246)	7 (6–22)	7 (6–22)
Resolved events, *n*	17	8	2	2
Fatal events, *n*	1	1	1	1
Leukocytosis				
Patients reporting event, *n*	5	1	6	4
Events, *n*	12	1	9	4
Event duration, days, median (range)	15 (4–107)	14 (14–14)	29 (1–1189)	37 (1–1189)
Resolved events, *n*	12	1	6	2

AP, accelerated phase.

CML, chronic myeloid leukemia.

CP, chronic phase.

* Includes the investigator verbatim term “myelosuppression.”
